# Are Rotator Cuff Tears in Anterior Shoulder Dislocation Associated With a High Critical Shoulder Angle? A Retrospective Analysis

**DOI:** 10.7759/cureus.103974

**Published:** 2026-02-20

**Authors:** Ameer Aldarragi, Niall Fitzpatrick, John M Ranson, Ronnie Davies, Chris Peach

**Affiliations:** 1 Trauma and Orthopaedics, Shoulder and Elbow Unit, Manchester University NHS Foundation Trust, Manchester, GBR; 2 Faculty of Science and Engineering, University of Manchester, Manchester, GBR

**Keywords:** critical shoulder angle, radiological screening, rotator cuff tear, shoulder dislocation, traumatic rotator cuff tear

## Abstract

Aims

This study aims to investigate whether the critical shoulder angle (CSA) could serve as an effective screening tool to predict acute rotator cuff tears in patients with first-time anterior glenohumeral dislocation and to assess the inter- and intra-observer reliability of CSA measurements in this cohort.

Methods

A review of all patients with a first-time anterior shoulder dislocation was carried out over a 17-month period at Manchester NHS Foundation Trust, across the Wythenshawe Hospital and Manchester Royal Infirmary sites. Three shoulder surgeons independently measured CSA on post-reduction radiographs, and all patients underwent imaging to assess for a traumatic rotator cuff tear (RCT). Statistical analysis was performed using unpaired t-tests and subgroup analysis based on age and rotator cuff tear presence. Inter- and intra-observer reliability was assessed using Cronbach's alpha.

Results

Sixty-five patients (36 female, 29 male) met the inclusion criteria. Forty-two patients (65%) had a rotator cuff tear detected on further imaging. The mean CSA for the tear group was 38.14° (SD 4.6), and for the non-tear group, it was 37.45° (SD 4.6), with no significant difference (p = 0.5598). Age was significantly higher in the tear group (64.5 years) compared to the non-tear group (58.1 years, p = 0.0225), with a positive correlation between age and the likelihood of sustaining a rotator cuff tear. Inter-observer reliability was found to be "good" (Cronbach’s alpha = 0.8519), while intra-observer reliability was "excellent" (Cronbach’s alpha ≥ 0.9282).

Conclusion

This study found no significant association between CSA and the incidence of rotator cuff tears following a traumatic anterior shoulder dislocation. However, increasing age was associated with a higher likelihood of sustaining a rotator cuff tear following anterior shoulder dislocation. This study has demonstrated that intra- and inter-observer reliability were excellent and good, respectively. These findings suggest that CSA may not be a reliable screening tool for rotator cuff tears following shoulder dislocation. Larger, standardised studies are needed to further explore this relationship.

## Introduction

The critical shoulder angle (CSA), first hypothesised by Moor et al., is a radiographic measurement of the inclination of the glenoid relative to the lateral extension of the acromion, measured as the angle between a line intersecting the glenoid and the anterior rim, to the lateral edge of the acromion. The role of anatomical variations in the predisposition of individuals to certain shoulder pathologies, including rotator cuff tear (RCT), has gained attention, with CSA emerging as a factor. CSA values >35° are said to be associated with RCT, and values of <30° are thought to be a predisposing factor for glenohumeral osteoarthritis. Pathology is believed to reflect mechanical load imbalance, particularly in abduction [1-2]. Amini et al. described a patient population in which 60% of patients had a CSA of less than 35° in the context of acute cuff tear, contrasting degenerative RCT in which the average CSA was 38° [3].

Traumatic dislocation of the glenohumeral joint is the most common joint dislocation, with an incidence ranging from 8.2 to 23.9 per 100,000 [4-5]. The incidence of RCT associated with anterior glenohumeral joint dislocation increases with age, with clinically relevant RCTs found in approximately 40% of those aged 40-60 years old following glenohumeral dislocation [6]. Surgical repair of those associated with instability has been shown to both decrease pain and improve patient satisfaction [7]. Recent studies have identified a correlation between larger CSA and re-tear or failure of repair, adding to the potential clinical benefit of accurate measurement [8].

Diagnosis of a clinically relevant RCT requires careful examination paired with radiological assessment. Clinical examination alone can lead to diagnostic uncertainty, with a systematic review by Hughes et al. demonstrating that the majority of clinical tests were inaccurate in the diagnosis of cuff pathology [9]. Clinical guidelines recommend the use of Magnetic Resonance Imaging (MRI) and Ultrasound scanning as a diagnostic tool, each of which has been shown to diagnose full-thickness cuff tears with a sensitivity and specificity of >92%, with lower accuracy for partial-thickness tears [6,10]. Radiological assessment allows operative planning and replicable calculation of CSA [11]. Both are time and resource-intensive, and in the case of MRI, have certain contraindications, such as the presence of certain metallic implants and electronic devices, such as a cardiac pacemaker, and as such, their use should be judicious.

The relationship between CSA and risk of traumatic RCT in those with first-time anterior glenohumeral joint dislocation is unknown. This could guide radiological resource management and decision-making when reviewing patients following their first-time shoulder dislocation. The author hypothesizes that higher CSA is associated with an increased likelihood of sustaining an acute traumatic RCT following first-time shoulder dislocation. Therefore, this study aimed to assess whether CSA could be used as an accurate screening tool to quantify the risk of acute RCT after the first time shoulder dislocation. In addition, the authors aimed to document the inter and intra-observer reliability of CSA in the patient cohort included in this project.

## Materials and methods

A retrospective analysis of the virtual fracture clinic database in Manchester NHS Foundation Trust, across the Wythenshawe Hospital and Manchester Royal Infirmary sites was performed through our electronic medical records (EMR) software between September 2022 and February 2024. Following the British Elbow and Shoulder Society (BESS) guidelines, those patients who are over 40 years old should be investigated early to rule out an RCT, whereas those patients under 40 are more likely to sustain labral tears [12]. Our inclusion criteria for the study were patients over 40 years old, first-time dislocations, no associated fractures, and no previous imaging to look for a cuff tear. Our exclusion criteria were patients <40 years old, known shoulder instability, previous stabilisation surgery, posterior dislocations, and no further radiological imaging to look for RCT following the dislocation episode. All patients within our study underwent an ultrasound scan conducted by an experienced musculoskeletal radiologist to assess for RCT. Our primary outcome measure was to assess if there was an association between CSA and traumatic RCT secondary to shoulder dislocation in patients over 40 years old. Our secondary outcome was to assess whether increasing age over 40 also increases the likelihood of sustaining an RCT post-anterior shoulder dislocation.

Three experienced shoulder and elbow surgeons within our hospital reviewed the post-reduction radiographs taken in the emergency department and calculated the CSA. The CSA was calculated as described in the original paper by Moor et al., which should be calculated on a true anteroposterior (AP) radiograph of the glenohumeral joint [1]. The surgeons were blinded to the presence or absence of RCT on ultrasound scan; they each reviewed the images independently and on two different occasions. The mean CSA was taken for statistical analysis using the unpaired Student's t-test. Categorical data were described by frequencies, and continuous data were summarised by the mean or median and minimum and maximum values. Patients were grouped based on whether further imaging showed an RCT or not. Subgroup analysis of patients in both groups based on age to look for an increased risk of an RCT as age increases using the unpaired student t test. Cronbach's alpha test was used to test for inter- and intra-observer reliability [13]. Significance level was set at p < 0.05. A power analysis was conducted to determine the minimum sample size required to detect a mean difference of 3° between groups, assuming a standard deviation of 4°, with a significance level of 0.05 and statistical power of 80%. The analysis indicated that 29 participants per group were required to detect the specified effect size. 

Bamigbola Jameelah (R0A), Manchester University NHS Foundation Trust, issued approval, with temporary registration number 270.

## Results

A total of 65 patients met the inclusion criteria of our study (36 female and 29 male). Forty-two patients (65%) presented with a rotator cuff tear following further imaging. Table 1 shows a summary of the patient characteristics. Mean age was 62 (range 42-80). There was no statistically significant difference between the mean CSA of patients in the tear group (38.14°, SD4.6) and in the no tear group (37.45°, SD4.6) (p = 0.5598). 

**Table 1:** Patient demographics between the tear group and no-tear group. Age is presented as mean (range). Continuous variables were compared using an independent-samples t-test (t = 2.20). Categorical variables were compared using the chi-square test (sex: χ² = 0.85; laterality: χ² = 0.15). A p-value < 0.05 was considered statistically significant.

Variable		Tear	No tear	P-value
No. of patients		42	23	-
Mean age (years)		64.5 (47-80)	58.1 (42-79)	P= 0.034
Sex	Male	21	8	p=0.358
	Female	21	15	
Laterality	Right	29	14	0.695
	Left	13	9	

Table 2 shows a subgroup analysis of the two groups comparing the different age groups. Overall, patients who presented with a rotator cuff tear had a higher age (64.5 years) compared to those who did not have a rotator cuff tear (58.1 years) (p = 0.0225). There was a positive correlation between tear incidence and increasing age; however, the subgroup analysis revealed no statistical significance between the groups. 

**Table 2:** Age sub-group analysis between the tear and no-tear groups Age is presented as mean (range). The mean age was compared using an independent-samples t-test (t = 2.20). Age sub-group distribution was compared using the chi-square test (χ² = 7.76, degrees of freedom = 3). A P-value < 0.05 was considered statistically significant.

Variable	Tear (n = 42)	No tear (n = 23)	P-value
Age, years (mean (range))	64.5 (47-80)	58.0 (42-79)	0.034
Age sub-group, n (%)			
<50	4 (9.5%)	6 (26.1%)	
50-59	11 (26.2%)	5 (21.7%)	
60-69	13 (31.0%)	7 (30.4%)	
≥70	14 (33.3%)	5 (21.7%)	0.051

In addition, we looked at the inter- and intra-observer reliability of the critical shoulder angle in our patient cohort. The Cronbach Alpha test was used to assess the consistency and reliability of the results between individual surgeons and comparing against other surgeons. Table 3 shows the results of the Cronbach's alpha test, which presents “excellent” intra-observer reliability. In addition, the Cronbach's alpha test revealed “good” results (0.8519) when looking at inter-observer reliability. 

**Table 3:** Cronbach's alpha test of intra-observer reliability.

Surgeon	Cronbach's alpha test	Interpretation
Surgeon 1	0.9310	Excellent
Surgeon 2	0.9282	Excellent
Surgeon 3	0.9938	Excellent

## Discussion

We had hypothesised that the CSA could be a useful screening tool in the assessment of risk of traumatic RCTs. Our study has shown that there is no association between CSA and RCT in the >40-year-old group, following traumatic anterior shoulder dislocation. Therefore, we cannot recommend this as a useful screening tool following traumatic shoulder dislocation to accurately predict whether patients have a rotator cuff or not to guide further radiological assessment. RCTs are common in the elderly population, especially following a traumatic injury [14]. This is the first study that assesses the CSA’s relation to the presence of RCTs following a traumatic anterior shoulder dislocation. The CSA has been reported to have an influence on the risk of RCT versus osteoarthritis of the glenohumeral joint, with an angle of <35° having an increased risk of osteoarthritis and >35° having an increased risk of RCT [1]. 

Age is a common risk factor for the onset of RCTs, with a higher incidence reported in the elderly population for both degenerative and traumatic tears [15,16]. It was observed in our study that there is a higher incidence of RCTs with increasing age following an anterior shoulder dislocation. From our study, we found that there was a positive correlation between increasing age and the likelihood of sustaining an RCT. There is a clear trend in the results that shows an increased likelihood of acute RCT following a dislocation for each decade of increased age. This is not statistically significant, but the authors suspect this represents a type 2 error in that with a large patient group, the difference would be significant. RCTs affect both the elderly and younger population; however, in the elderly group, there can be a higher incidence of asymptomatic tears. Traumatic tears in the elderly are recommended for early surgical repair to prevent tendon retraction and improve post-operative outcomes [17,18]. Therefore, early detection and prompt management are beneficial in optimising successful surgical outcomes. 

As described by Moor, the CSA is affected by the rotational position of the scapula on the radiograph. In their study, they reported that a reproducible CSA with a variability of ≤ 2° was achievable despite malrotation of radiographs of up to 20° [1]. The projections on which the radiographs were taken for the patients analysed in this study were variable. Some patients did have a true AP projection of the glenohumeral joint; however, others had more rotated views. This is due to the radiographs being taken following reduction of the anterior dislocation, and therefore, the patient is likely to be experiencing pain whilst the radiographs are being taken. We were unable to comment on the rotation of the scapula from the radiographs analysed, and therefore the effect of the radiograph quality on the CSA calculation remains unknown. Radiographs taken in the clinic following the early recovery period may allow more accurate measurement of the CSA due to the resolution of pain and the ability to capture a true AP radiograph of the shoulder joint. 

The CSA is reported to be reliable and reproducible in the literature, especially in terms of interobserver reliability. Rojas et al performed a meta-analysis looking at 14 studies comparing the reliability of CSA on their test subjects [19]. However, their study had a very high heterogeneity owing to the methodologies of the studies analysed and their sample size. Furthermore, CSA has been shown to be susceptible to variation in calculation when the scapula line is changed, which is shown in the variation from a true AP image of the glenohumeral joint. This has been shown to reduce the reliability and reproducibility of CSA with non-true AP images vs true AP images [20]. This was highlighted by the senior author, who rated the radiographs based on the true AP projection of the films analysed, which highlighted that only 31% of the radiographs in our study appeared to be true AP projection films. This could have led to a reduced reliability in the inter- and intra-observer groups; however, this was not a noticeable error in our study. On the other hand, a recent study by Schiefer et all looked at the inter- and intra-observer reliability of CSA measurements using both X-Ray and MRI images. This showed that the CSA measured on both X-ray and MRI were the same (P<0.001) [11]. In this study, the radiographs taken were shown to be true AP images of the glenohumeral joint, which likely contributed to their highly reproducible CSA measurements in both imaging modalities. 

The effect of CSA on glenohumeral instability has been studied in the literature, and studies have shown a positive correlation between CSA and vertical shear on the shoulder joint, ultimately leading to joint instability. Viehöfer et al studied a three-dimensional computed model of the glenohumeral joint to assess instability for higher CSA associated with an RCT versus a normal CSA. They found that patients with a higher CSA projected much higher shear forces and reduced compressive forces from the deltoid when compared to a shoulder joint with a normal CSA [21]. The findings of this study are in agreement with a more recent study by Oswald et al., which showed that an increased CSA was associated with increased vertical instability in abduction, and in addition, a lower CSA was associated with increased instability in flexion position [22]. Interestingly, both groups of patients that we have included in our study (tear vs no tear) had a higher than average CSA, predisposing them to an RCT. However, the correlation with increased angle and the presence of RCT could not be proven from our study. Having a higher CSA could be a predisposing factor for instability of the glenohumeral joint, and therefore, those patients would present with a dislocation following a minor trauma. However, this observation would need further assessment on a bigger scale, with a control and test group to assess this relationship. 

Our study has some limitations. Firstly, the data collected was not intended for the calculation of the CSA, and therefore, deviation away from a true AP radiograph may have contributed to variation in CSA analysis. Secondly, not all patients during the study period with shoulder dislocations were followed up with further imaging to assess for an RCT, which might have led to bias. Finally, our sample size in the no-tear group is smaller than the power calculation. A larger sample size would have increased the power of the study to identify other associations. 

## Conclusions

From our study, we conclude that there is no significant difference in the CSA of patients presenting with or without a rotator cuff tear following a traumatic anterior glenohumeral joint dislocation. An increase in age was observed to be associated with an increased likelihood of sustaining a rotator cuff tear following glenohumeral joint dislocation. Both inter and intra-observer reliability of CSA in our study was high. Further study of this association with larger patient numbers and standardised radiograph techniques is required to study the association further.
